# Animal Models of Intervertebral Disc Diseases: Advantages, Limitations, and Future Directions

**DOI:** 10.3390/neurolint16060129

**Published:** 2024-12-09

**Authors:** Jin Young Hong, Hyunseong Kim, Wan-Jin Jeon, Changhwan Yeo, Hyun Kim, Junseon Lee, Yoon Jae Lee, In-Hyuk Ha

**Affiliations:** Jaseng Spine and Joint Research Institute, Jaseng Medical Foundation, Seoul 135-896, Republic of Korea; vrt23@jaseng.org (J.Y.H.); biology@jaseng.org (H.K.); cool2305@jaseng.org (W.-J.J.); duelf2@jaseng.org (C.Y.); khyeon94@jaseng.org (H.K.); excikind@jaseng.org (J.L.); goodsmile@jaseng.org (Y.J.L.)

**Keywords:** animal model, lumbar disc herniation, lumbar disc degeneration, lumbar spinal stenosis, advantages of animal model, limitations of animal model, future directions

## Abstract

Animal models are valuable tools for studying the underlying mechanisms of and potential treatments for intervertebral disc diseases. In this review, we discuss the advantages and limitations of animal models of disc diseases, focusing on lumbar spinal stenosis, disc herniation, and degeneration, as well as future research directions. The advantages of animal models are that they enable controlled experiments, long-term monitoring to study the natural history of the disease, and the testing of potential treatments. However, they also have limitations, including species differences, ethical concerns, a lack of standardized protocols, and short lifespans. Therefore, ongoing research focuses on improving animal model standardization and incorporating advanced imaging and noninvasive techniques, genetic models, and biomechanical analyses to overcome these limitations. These future directions hold potential for improving our understanding of the underlying mechanisms of disc diseases and for developing new treatments. Overall, although animal models can provide valuable insights into pathophysiology and potential treatments for disc diseases, their limitations should be carefully considered when interpreting findings from animal studies.

## 1. Introduction

Spinal stenosis, herniated disc, and degenerative disc disease are common spinal disorders that predominantly occur in the lumbar region and are closely associated with aging. These conditions often lead to symptoms such as pain in the neck, back, arms, legs, hands, or feet, as well as numbness, tingling, and muscle weakness [1,2,3]. Spinal stenosis involves a narrowing of the spinal canal in the lower back, compressing the spinal cord, nerve tissues, and cerebrospinal fluid, and is the leading reason for spinal surgery in patients over 65 years of age [4]. A herniated disc results from the deterioration of disc structure, causing the nucleus pulposus (NP) to protrude and place pressure on the spinal cord, nerve roots, or vertebrae, leading to radicular pain and weakness; over 90% of cases occur at the L4–L5 or L5–S1 disc spaces. Degenerative disc disease is a natural consequence of aging, where the discs cushioning the vertebrae wear down and shrink, resulting in lower back pain and potentially progressing to conditions like herniated disc, spinal stenosis, or arthritis [5,6]. These disorders are particularly prevalent in the lumbar region, making them clinically significant and critical targets for treatment development. However, the underlying pathophysiology of these conditions remains poorly understood, and despite the availability of various treatments, their success rates are limited. Animal models have been used extensively to elucidate the underlying mechanisms and potential therapies for intervertebral disc (IVD) diseases. These models provide valuable insights owing to their ability to mimic human conditions, cost-effectiveness, and control of experimental variables; however, their use also has some limitations. The major limitations are the anatomical and physiological differences between animal and human spines, the subjective nature of pain assessment, and ethical considerations of using animals for research. Future directions include the development of more relevant animal models, such as those of nonhuman primates and genetically modified rodents, and the use of non-invasive imaging techniques to monitor disease progression. This review explores the strengths and limitations of using animal models for disc diseases and outlines future directions to enhance their translational relevance. By offering a comprehensive overview of the current state of these models, this review aims to support future research in the field and contribute to the development of more effective treatments for these challenging conditions.

## 2. Animal Models of Disc Diseases

### 2.1. Laboratory (Small) Animal Models for Lumbar Spinal Stenosis

**Figure 1:**
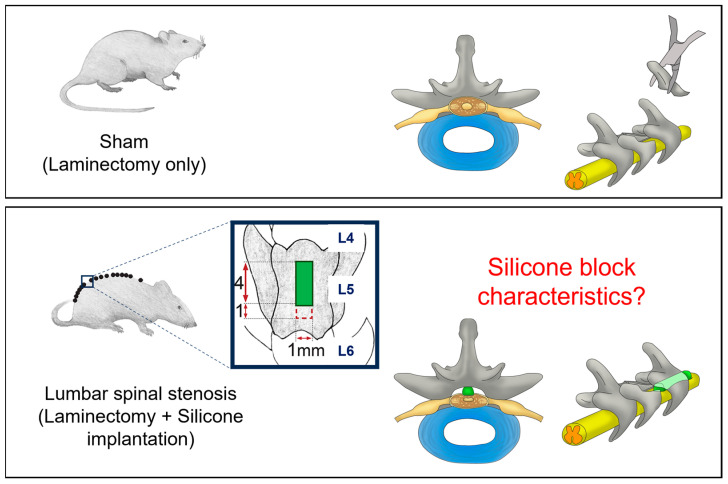
An image illustrating the creation method of the LSS rat model. After laminectomy, a custom-made silicone block is inserted into the spinal canal to compress the nerves, inducing LSS symptoms. However, evaluations of the silicone block’s properties, such as tensile strength, yield strength, elastic modulus, corrosion, creep, and hardness, are insufficient. Adapted with permission from ref. [13].

Lumbar spinal stenosis (LSS) is a common degenerative disorder that affects the lower spine. It involves the narrowing of the spinal canal, which can compress the spinal cord and nerves, leading to symptoms such as pain, numbness, and weakness in the lower back, buttocks, and legs [7]. Animal models of LSS have been developed to study the pathophysiology and potential treatments of this condition [8,9,10,11,12]. A commonly used animal model of LSS is the rodent model. The surgical technique for creating this model involves performing a laminectomy to expose the spinal canal and inserting a custom-made silicone block into the canal to induce spinal canal narrowing. The silicone block gradually compresses the spinal cord and nerves, leading to symptoms similar to those of human patients with LSS. However, most studies have not characterised the silicone blocks used to mimic a narrowed spinal canal. The properties of the silicone blocks, such as tensile strength, yield strength, elastic modulus, corrosion, creep, and hardness, should be carefully studied (Figure 1) [13].

Most studies have used block-type silicone, chiefly of the dimensions of 4 mm (length) × 1 mm (width) × 1 mm (height) [13,14,15,16,17,18,19,20,21]. A study that used sheet-type silicone to create an LSS model reported only the thickness of the silicone piece (0.51 mm), with no information on its length, width, or characteristics [22]. In most studies, stenosis was induced by inserting one silicone block into the spinal canal between the L4 and L6 levels [13,14,15,16,17,18,19,20,22,23,24,25,26,27]. 

**Figure 2:**
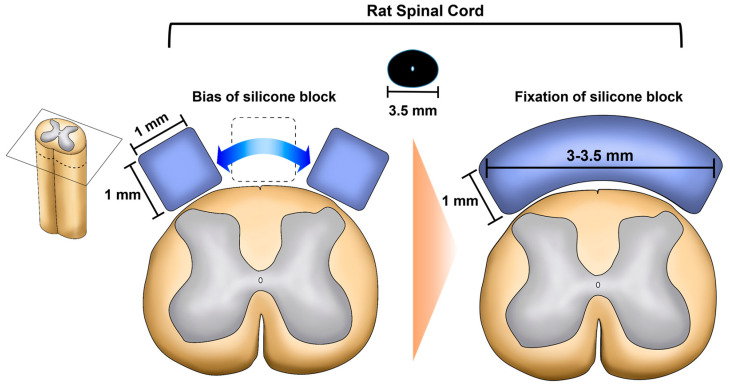
A schematic showing improvements in the size and shape of the silicone block designed to induce uniform spinal stenosis. In the left image, the current 1 × 1 mm^2^ square-shaped silicone block is prone to lateral displacement, limiting uniform histological changes and symptom induction. To address this, the right image proposes using a wider silicone block, approximately 3–3.5 mm to match the spinal cord width, allowing it to remain centrally positioned and enabling more consistent symptom induction.

However, the insertion of two silicone blocks into two spinal canals at the L4, L5, or L6 levels has also been reported [20,21,22]. Stabilising the silicone block in the central position on the dorsal side of the spinal cord is difficult owing to the size of the block. The position of the block is biased to the right or left, preventing uniform nerve damage on both sides. The spinal cord appears oval in the axial view, and its width is approximately 3.5 mm. Even if a 1-mm wide silicon block is inserted at the centre, it is likely to be biased to one side due to the continuous movement of the rat. Therefore, the width of the silicone blocks should be approximately 3–3.5 mm, similar to the width of the spinal cord in rats, to induce uniform symptoms and nerve damage bilaterally (Figure 2).

Further, the hardness of the silicone blocks has the greatest impact on the compression of the spinal cord with regard to locomotor function. A study comparing silicone blocks of different hardness used to induce spinal stenosis showed that a silicone block hardness of 70–90 kPa could provide an index for creating LSS models in rats with mild, moderate, or severe LSS [13]. The various conditions for generating spinal stenosis in rats using silicone blocks are summarised in Table 1.

Hypertrophy of the ligamentum flavum (LF) is a major cause of LSS, which narrows the spinal canal and exerts pressure on the spinal cord or nerves, causing back or leg pain. Under normal physiological conditions, the LF provides strong support to the spine and prevents excessive movement of the lower back; however, its thickness and stiffness increase with age. Park et al. found that patients with LSS had an average LF thickness of 4.44 mm (range: 3.4–5 mm), which was significantly greater than the 2.44 mm thickness observed in healthy individuals without LSS or hypertrophy [28]. Sairyo et al. reported that hypertrophy of the LF develops with age and occurs mainly in the L3/4 and L4/5 lumbar regions [29]. Therefore, an animal model that reflects the major causes of LSS, such as LF hypertrophy, may increase the possibility of clinical translation. Wang et al. established a novel rat model of LSS by inducing LF hypertrophy; they completely resected the L5 and L6 spinous processes and performed semi-grinding of the bilateral L5/6 facet joints with a grinder and a round drill bit, which resulted in increased lumbar motion and subsequent LF hypertrophy [30]. Although the authors suggested that their method was relatively simple, rapid, and safe for mimicking the pathological features of LF hypertrophy-induced LSS observed in humans, they did not standardise the method for semi-grinding of the bilateral L5/6 facet joints to induce LF hypertrophy. It is critical to improve the reproducibility of preclinical animal studies. Therefore, the grinding time, speed (rpm), and drill bit size should be specified to induce uniform biomechanical stress on LF.

### 2.2. Large Animal Models for Lumbar Spinal Stenosis

Large animal models, particularly pigs, rabbits, and dogs, play a critical role in spinal disease research owing to the anatomical and physiological similarities between their spines and the human spine. These models are relatively understudied compared with rodent models but make significant contributions by mimicking the pathological conditions of LSS and replicating the clinical symptoms. Studies using pig models have investigated the impact of structural changes in the spine on spinal function. For instance, research analysing the effects of laminectomy and partial facetectomy procedures, commonly performed to relieve spinal canal compression, has revealed that such surgeries may increase the risk of shear force-related spinal fractures in patients [31]. Another study assessed the effectiveness and safety of a newly developed interspinous process spacer for the treatment of posture-dependent lumbar spinal stenosis, suggesting the potential of this technique as a minimally invasive surgery [32].

Rabbit models have been used to replicate the pathological changes considered the primary cause of LSS by inducing LF hypertrophy and fibrosis through increased mechanical stress. Specifically, methods such as resection of the L3–L4 supraspinal muscle and posterolateral fusion at the L2-3 and L4–L5 levels with metal implants and titanium locking screws were employed to elevate mechanical stress at the L3–L4 level [33,34,35]. These models have elucidated the role of molecules like Wingless-Type MMTV Integration Site Family, Member 1 (WNT1) inducible signalling pathway protein 1 (WISP-1), which are upregulated by mechanical stress and promote inflammation and fibrosis in the LF [34]. Additionally, these models have been used to evaluate the efficacy of therapeutic interventions, such as the impact of cyclopamine on reducing LF fibrosis, contributing to the development of new treatment strategies. In another study utilising a rabbit model, two 3-F Fogarty catheters were inserted beneath the L6 plate and anchored to the spinous process of the fifth lumbar vertebra. LSS was induced by gradually inflating the Fogarty catheter balloon with air until a myelographic block was achieved at the L6 level, which was confirmed using fluoroscopy [36]. The method for inducing LSS in rabbits is summarized in Table 2.

Research conducted with large animal models provides a deep understanding of the pathological changes associated with LSS and serves as foundational data for the development of future therapeutic strategies. Particularly, these models precisely replicate clinical symptoms related to nerve compression and play a crucial role in evaluating the effectiveness of therapeutic interventions. Thus, large animal models are an essential research tool for identifying new molecular targets for the prevention and treatment of LSS and have the potential to lead to the development of treatments that improve the quality of life for patients. Future research should utilise a variety of large animal models to more accurately replicate and analyse the complex symptoms of LSS.

### 2.3. Laboratory (Small) Animal Models for Lumbar Disc Herniation

Lumbar disc herniation (LDH) refers to the bulging, slipping, or rupturing of an IVD. It occurs when the cushion-like soft NP of a lumbar disc protrudes from the spinal canal through a tear or weak region in the tough outer covering [37]. This can cause compression of the surrounding nerves, leading to pain in the lower back, which may radiate to the buttocks, thighs, and legs; numbness or tingling in the legs or feet; weakness in the legs; and difficulty standing or walking [38]. However, the exact cause of LDH is not always clear but may be related to aging, genetics, repetitive lifting or twisting motions, or injury to the back [39,40]. Therefore, it is crucial to study the underlying mechanisms of LDH through preclinical studies to inhibit disease progression and improve outcomes in human patients.

Animal models of LDH have primarily involved rats. The traditional surgical procedure for creating an LDH rat model involves the placement of a small incision in the back of the rat and exposure of the lumbar nerve roots and dorsal root ganglion (DRG), mostly at L4–L6 levels, through a laminectomy. Autologous NP harvested from coccygeal IVDs is transplanted into the exposed DRG or lumbar nerve roots proximal to the corresponding DRG without mechanical compression [41,42,43,44,45,46,47,48,49,50,51,52,53,54,55,56,57,58,59,60,61,62,63,64,65,66,67,68,69,70,71,72,73,74,75,76,77,78,79,80,81,82]. However, the mechanical pressure applied to the nerves through this method can vary between studies. Most reports do not mention the amount of NP used, while a few reports have specified the use of 5 or 10 mg NP for transplantation [51,52,57,68,70,71,72,80,81]. Further, the extent of mechanical pressure to the nerve and biochemical changes, including degeneration and inflammatory response, has not been studied in relation to the amount of NP used. The prolapse of the NP precedes degenerative changes, and the activity of proinflammatory cytokines and chemokines in the NP increases with the progression of degenerative changes. When the NP with increased inflammatory activity escapes into the epidural space, the inflammatory response increases rapidly through the mobilisation of immune cells, resulting in neurotoxicity and pain [83]. Therefore, an NP transplantation model with increased inflammatory activity should be constructed to obtain a model that simulates clinical conditions. In addition, NP is a mucous protein chiefly containing water [84] and cannot be easily stabilised on the DRG and nerve roots. Kim et al. found the autologous transplantation of the IVD NP challenging due to partial loss and poor stabilisation at the recipient site [85]. These limitations indicate the challenges associated with the precision and reproducibility of animal models. Further, no study has confirmed changes over time in transplanted autologous NP to date. Clinically, LDH refers to the disease where the NP separates, hardens, and presses on the nerve in a state where it resembles a bone. However, as pain may be induced not only in the presence of mechanical compression but also in its absence, it is highly possible that, in addition to mechanical pressure, biochemical mechanisms, including the inflammatory response, are also involved in the pathogenesis of neuromuscular lesions.

**Figure 3:**
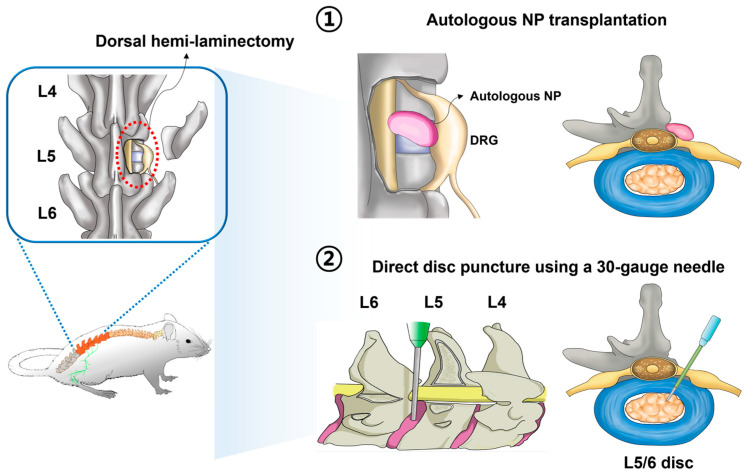
An image illustrating the representative methods for creating LDH rat models. The first method involves harvesting autologous NP from the coccygeal IVD and implanting it near the lumbar nerve root adjacent to the DRG. The second method involves exposing the L5–L6 discs, performing a direct disc puncture, and simultaneously injecting the pro-inflammatory cytokine IL-1β. Adapted with permission from ref. [85].

It has been shown that the herniated IVD naturally resorbs over time and decreases in size [86,87]. LDH is a nonsurgical disease that can be treated without surgical decompression in many patients if the inflammatory response is first suppressed and active rehabilitation is performed. However, a few studies have examined the mechanism of conservative treatments where the NP leak through the annulus fibrosus (AF) is naturally reabsorbed. Recent studies have reported that extensive crosstalk between NP and macrophages (M1 or M2) may play an essential role in triggering NP self-absorption [88]. Therefore, LDH should be classified based on morphological differences in IVD herniation by developing LDH animal models that reflect the various clinical aspects and examining how the effects of conservative treatment differ. In addition, it has been reported that most autologous NP transplantation rat models experience allodynia and hyperalgesia 1 day postoperatively, which decreases within a few weeks. Clinically, most patients with early acute neuropathic radiation pain improve within several weeks to months; however, some patients may continue to experience pain [89]. Therefore, it is necessary to develop a model with long-term follow-up of parameters of radiating neuropathic pain. Kim et al. proposed a novel LDH model where the proinflammatory cytokine interleukin 1 beta (IL-1β) was injected simultaneously with direct disc puncture to more closely reflect the clinical pathophysiological environment in L5–L6 discs exposed through hemilaminectomy. Notably, their proposed method generated LDH models of differential severity, based on three concentrations (1, 10, or 100 ng/100 μL) of injected Interleukin-1 beta (IL-1β)) [85]. Although their new method of LDH model creation was evaluated using various approaches to investigate the degree of histological damage, inflammation, degeneration, neuropathic pain through immunohistochemistry, gene expression associated with inflammation and pain, and in vivo functional assessment, the course of the NP was not observed for a long time. The axial magnetic resonance imaging (MRI) image only showed a reduced amount of NP, and the sagittal view did not show that the protruding NP had hardened and compressed the nerve. A schematic diagram illustrates the surgical differences between the two main methods for creating LDH rat models (Figure 3) [85], and studies that established LDH models using autologous NP transplantation are summarized in Table 3.

### 2.4. Large Animal Models for Lumbar Disc Herniation

In studies on LDH using pigs, models were developed to mimic the mechanical and chemical damage mechanisms of disc herniation. These models were then used to evaluate the cerebrospinal fluid (CSF) for changes in biomarkers related to nerve tissue damage, inflammation, and pain. The method of inducing disc herniation in these models was similar to that used in rodent models, involving autologous NP transplantation. The NP was harvested from the L2–L3 discs of pigs using a retroperitoneal approach, and approximately 100 mg of the extracted NP was placed on the left side of the S1 nerve root while also applying mechanical compression using an ameroid constrictor. Seven days after inducing disc herniation, a laminectomy from L4 to S3 was performed, exposing the dural sac, and 6 mL of CSF was collected from L4 to L5 using a syringe with a 0.8-mm bent needle. Significant increases in neurofilament and nociceptin concentrations were observed in the groups with mechanical compression or mechanical compression and NP was compared with the sham surgery group, confirming changes in CSF biomarkers [90].

In canine experimental models, the application of NP was modelled to experimentally induce thrombosis formation around the nerve root, decrease blood flow, and reduce nerve conduction velocity. This study evaluated the effects of 5-Hydroxytryptamine 2A receptor (5-HT2A) receptor antagonists on these changes, exploring the potential for improving nerve blood flow in patients with LDH. After performing a partial laminectomy on the tail side of the sixth lumbar vertebrae, the dorsolateral portion of the L6/L7 IVD was incised with an 18-gauge needle to allow the NP to flow out toward the abdominal side nerve root. Following the administration of 5-HTRA, an increase in vessel diameter and blood flow was observed; however, this was limited to inflamed nerve roots and not observed in uninjured nerve roots. This suggests that 5-HTRA may be a potential agent for improving blood flow in the nerve roots of patients with LDH [91]. Another study involved injecting recombinant human matrix metalloproteinase 7 (rhMMP-7) into the intervertebral discs (L2–L3, L3–L4, L4–L5) of dogs to investigate its effects on the degradation of human herniated discs and its impact on surrounding tissues. The injection of rhMMP-7 into the disc reduced proteoglycan and water content and increased blood keratan sulphate levels while not affecting the injection site or nerve tissue. This suggests that rhMMP-7 has potential as a new alternative for chemical nucleotomy in the treatment of lumbar disc herniation, with fewer complications expected than existing drugs [92]. Experimental studies exploring the impact of age on the pathological events associated with disc herniation also utilised canine models. Beagle dogs in three age groups—6 months, 12 months, and 24 months (equivalent to 10, 15, and 24 human years)—underwent laminectomy and discectomy at L4–L5, L5–L6, and L6–L7, and autologous IVD fragments were placed around the nerve root. The study results showed differences by age, with neovascularisation, lymphocytes, macrophages, and fibroblast infiltration observed only in the 24-month age group around the NP fragments. Degenerative changes in nerve root fibres were also observed only in this group. In contrast, nerve root fibres in the control and AF groups were normal in all age groups. This study suggests that the inflammatory response and nerve root damage caused by the herniated disc may vary with age, with neuroprotective mechanisms present in younger animals and more pronounced inflammation and absorption changes around the NP fragments in older animals. This finding indicates that age should be an important consideration in developing treatment strategies for LDH [93].

Rabbit studies evaluated the effects of midkine, lipopolysaccharide, and basic fibroblast growth factor on the natural absorption process of disc herniation [94,95,96]. These studies used an anterior retroperitoneal approach to harvest the L1–L2 IVD and transplanted it into the posterior dural space of the L4 vertebra in rabbits. These studies focused on exploring new methods to accelerate the absorption of herniated discs through relocation to the dural space. Methods for creating LDH models using large animals, including pigs, dogs, and rabbits, are summarized in Table 4.

Therefore, the use of large animal models, such as pigs, dogs, and rabbits, helps in evaluating various treatment strategies for IVD herniation. This contributes significantly to the development of diagnostic and treatment methods that can be applied to human patients. Moreover, a deeper understanding of the resorption mechanisms of disc herniation can provide opportunities to discover new therapeutic targets, ultimately leading to the development of innovative treatments that can be applied to human patients.

### 2.5. Laboratory (Small) Animal Models for Lumbar IVD Degeneration

**Figure 4:**
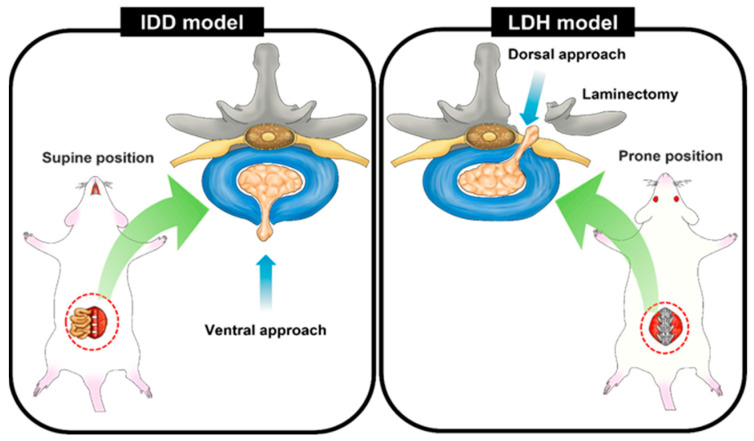
A schematic illustration highlighting the fundamental differences in creating IDD and LDH rat models at the lumbar level. The IDD model involves a ventral approach where a needle is used to puncture the lumbar disc, inducing gradual disc degeneration. In contrast, the LDH model employs a dorsal approach to penetrate the AF surface, inducing direct NP extrusion without damaging peripheral nerves or the DRG, or simulates the protruded state by mimicking disc herniation through NP autografting at the site.

IVD degeneration (IDD) is a common age-related disorder of IVD that can lead to LBP and other spinal conditions [97]. The IDD animal model is developed by piercing the IVD, usually through a ventral approach using a needle, to create a controlled injury that results in disc degeneration over time. The pathology of IDD differs significantly from that of LDH, where intradiscal NP escapes through the gap in the outer layer of the AF into the spinal cord, nerve root, and DRG, compressing the surrounding nerves and stimulating nerves through an inflammatory response [98]. In contrast, IDD occurs when the height between the vertebrae decreases; the IVD becomes thinner as degeneration progresses, and the disc that serves as a cushion between the vertebrae does not function properly [99,100]. Clinically, chronic LBP alone does not cause lower extremity pain. Therefore, a fundamental difference exists in constructing LDH and IDD animal models. The most significant difference lies in the approach direction used to induce disc damage (Figure 4).

In LDH animal models, laminectomy is performed by incising the skin on the back; however, in IDD animal models, laminectomy is unnecessary because the disc is exposed through a ventral approach after placing an abdominal skin incision. Therefore, the prolapse of the NP is directed toward the organ rather than the nerve, and the IVD is thinned to artificially reduce the height between vertebrae.

Although animal models of IDD are mainly produced in rodents and can be performed on cervical, coccygeal, or lumbar IVDs, degenerative IVDs in the lumbar region are particularly relevant for evaluating chronic back pain and gait disturbances caused by degenerative changes for several reasons. Studying the degenerative IVDs in this specific region enables researchers to better mimic the clinical scenario and understand the underlying mechanisms. Humans primarily experience chronic back pain and gait disturbances in the lumbar region due to degenerative changes in IVDs [101]. Researchers can better mimic the pathophysiological processes and symptoms observed in humans using animal models with degenerative IVDs in the lumbar region. This enables a more accurate assessment of potential treatments and interventions for chronic back pain and gait disturbances in clinical practice.

A PubMed search of reports published in the last 5 years (from 2019 to the present) using the search term “disc degeneration rat” showed that IDD models were developed at the lumbar level in 44 studies out of approximately 581 studies. Most studies developed degenerative models at the coccygeal level in rats (Co5–6, Co6–7, Co7–8, and Co8–9). Studies have described the preparation of an IDD model at the lumbar level through a ventral approach to induce NP prolapse; the thickness of the needle used when puncturing the disc ranged from 18 to 27 gauge, and the insertion depth of the needle was approximately 2 to 4 mm. Methods for inducing disc degeneration include the delayed insertion of a needle, repeated puncture of the same site over time, puncturing not only the midline of the disc endplate but also its right and left aspects, and simultaneously removing the NP.

**Figure 5:**
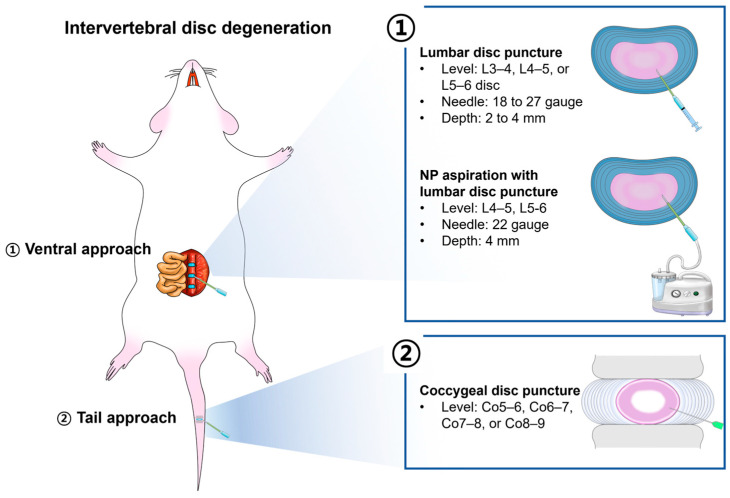
An image illustrating representative methods for generating IDD models in rats. The first method involves creating lumbar IDD by performing a needle puncture on the lumbar IVD using an 18- to 27-gauge needle through a ventral approach. Another approach for inducing IDD involves directly aspirating the NP from the lumbar disc through disc puncture. Most methods induce IDD by targeting the coccygeal region through a disc puncture approach via the tail. Adapted with permission from ref. [102].

The schematic illustrates the surgical differences among three representative methods for creating an IDD rat model (Figure 5) [102].

The studies that produced the IDD model in lumbar level with ventral disc puncture are presented in Table 5. Some studies have been performed with dorsal or dorsolateral surgical approaches to fabricate an IDD model [103,104,105,106,107,108,109,110,111].

Regarding the validity of the therapeutic candidates applied in IDD models produced by various methods, verifying whether they are effective in relieving chronic LBP is important. However, no standardised method exists to confirm relief from chronic LBP. In some studies, the increase in immobility time observed during the tail suspension test was attributed to chronic LBP, and the test was presented as an LBP evaluation method [102,141,142]. However, immobility under unavoidable stress in a short period has mainly been used to assess depression-like behaviour or behavioural despair [143]. In addition, one study noted that rearing observations, specifically of the total number of rearing and the duration of rearing behaviour with hindlimb are approaches for LBP behaviour [132]. Therefore, an evaluation method to directly examine chronic LBP should be established; this method should be independent of other variables and assess behavioural changes that can result only from LBP.

Additionally, most experimental studies on the IDD model have performed the Von Frey test. The Von Frey test is a neurophysiological test used to determine the mechanical pain threshold by applying a mechanical stimulus to the sole of the foot [144]. Although the Von Frey test is not an accurate method for evaluating radicular pain, most studies have shown a valid difference in decreasing latency in the IDD model. As mentioned earlier, most patients with degenerative discs rarely experience pain radiating to the lower limbs, and most experience LBP. Therefore, it is necessary to develop animal models that reflect these clinical symptoms. Hong et al. proposed a model where the NP was aspirated by perforating the disc using a ventral approach to create an IDD model [102]. They argued that the model reflected the clinical symptoms of a patient with degenerative disc with only chronic LBP and no radiating pain, based on the evidence that there was no significant difference in the Von Frey test results compared with that in the sham group. However, another study using an IDD model prepared through disc puncture and NP aspiration reported that the Von Frey test showed a significant difference between control (sham or naïve) and IDD rats from 7 days with a decrease in the withdrawal threshold up to 28 days postoperatively [120]. The results may differ based on the method used for the Von Frey test (automatic measurement method using equipment or the manual measurement method using Von Frey filament), the equipment used for NP suction, suction pressure, and time. Therefore, a more consistent and standardised model that reflects the clinical symptoms of patients with disc degeneration should be developed.

### 2.6. Large Animal Models for Lumbar IVD Degeneration

In degenerative disc studies using pigs, the primary focus was on the lumbar regions L1–L2, L3–L4, or L4–L5. Here, 16- to 20-gauge needles, approximately 3.5 mm drill bits, or scalpel blades were employed to perforate or excise the AF and aspirate the NP, a thereby inducing degenerative changes in the discs [145,146,147,148,149,150,151,152,153,154]. The pig models of degenerative disc created through this method were used to assess the effects of mesenchymal stem cell injections on degenerative changes and regenerative treatments [148,150]. Additionally, these models contributed to understanding the pathological changes in degenerative disc tissue by immunohistochemically analysing the expression of vascular endothelial growth factor receptor in the degenerative disc tissues [153]. The spine of pigs, sharing similar structural and biological characteristics with the human spine, proved to be an effective model for studying IDD and the effects and implications of discography. Discography performed in pigs, which mirrors the procedure used in humans, involves the injection of contrast material into the IVD followed by evaluation of the disc’s condition using X-ray or MRI. These pig model discography studies play a crucial role in understanding human IDD diseases and studying the biological changes and pressure variations in adjacent discs during the degeneration process [147,154].

Similarly, canine models of degenerative disc employed needles and blades for AF perforation and incisions, and NP aspiration in discs located at L1–L2, L3–L4, L4–L5, L5–L6, or L6–L7 [155,156,157,158]. Studies evaluated whether the controlled release of corticosteroids in biological materials could prolong their presence, providing pain relief and improving degenerative changes over an extended period without the adverse effects typical of high bolus doses. Results showed that the continuous local release of triamcinolone acetonide (TAA) did not impact the disc height index or other radiological parameters; however, low-dose TAA microspheres were found to reduce the immunopositive response of nerve growth factor in degenerative NP tissues [157]. Another study evaluated the efficacy of nucleus pulposus cells (NPCs) delivered via recombinant adeno-associated virus (rAAV)–human telomerase reverse transcriptase (hTERT) in a canine model of disc degeneration. This study demonstrated that NPCs delivered via rAAV-hTERT were superior in delaying the degeneration process and preserving structural integrity, extracellular matrix content, and mechanical stability in the canine model compared with NPCs [158]. Another method used to induce lumbar disc degeneration in dogs involved attaching coil springs to the lumbar IVD for up to 53 weeks. The study showed that long-term compression did not induce visually detectable degeneration in the canine discs, though subtle changes and numerical alterations in proteoglycans and collagen were observed [159].

Rabbit models of degenerative disc primarily utilised 16- or 18-gauge needles to perforate the AF and induce disc degeneration. The depth and frequency of the perforation varied depending on the research objectives, and some studies included invasive procedures such as ligation, removal or aspiration of the NP, or implantation of devices to apply mechanical stress to the IVD. Studies involving the chemical induction of disc degeneration in rabbits injected chemicals such as chymopapain to induce degeneration, mimicking the biochemical aspect of disc degeneration. Techniques such as direct gene transfer were also used to alter gene expression in disc cells, thereby inducing degenerative changes.

The monkey model is a valuable animal model for studying IDD due to its spinal structure and biochemical characteristics that are similar to humans [160]. In particular, studies on Cynomolgus, Rhesus, and Macaque monkeys have shown that the morphological characteristics of the cervical, thoracic, and lumbar regions, as well as the distribution of intervertebral disc components (proteoglycans and collagen) and degenerative changes, are similar to those in humans, demonstrating the structural similarity between monkey and human spines [161,162,163]. Furthermore, non-invasive techniques such as T1ρ magnetic resonance imaging (T1ρ MRI) have proven to be effective in assessing early disc degeneration [164]. Studies in female Macaque monkeys have identified an IDD pattern similar to that seen in human females, suggesting that this model could contribute to understanding the pathological mechanisms of IDD and developing treatment methods [165]. Various studies have evaluated the monkey model as a useful tool for understanding the structural and pathological mechanisms of human spinal diseases, as well as for research on and the diagnosis of disc degeneration. Experimental methods to induce IDD included disc puncture using 15- or 20-gauge needles in Rhesus monkeys or the injection of bleomycin or pingyangmycin solutions into the lumbar regions (L3/4, L4–L5, and L5–L6) to induce mild and progressive degeneration [166,167,168]. These models provide valuable data for human IDD research and are particularly useful for studying the pathological mechanisms and potential treatments for early disc degeneration.

Studies using sheep as a model for spinal disc research provide essential foundational data for understanding the pathophysiology and developing treatments for human spinal disorders, particularly IDD. Sheep share similar characteristics with humans in terms of spinal size, biomechanics, and histological composition, making them a commonly used model in spinal research [169]. While sheep lumbar intervertebral discs differ slightly from human discs in size and thickness, the structure of the AF and NP is comparable to that of humans. Biomechanically, sheep discs also exhibit similar compression strength and elasticity, making them suitable for degenerative disc research [170]. Although sheep are quadrupeds and differ from humans, who are bipedal, the mechanical stresses exerted on the spine due to body weight and posture show similar patterns. Degenerative disc models in sheep are typically created by inducing disc injury using a drill bit or a needle puncture [171,172]. The drill bit model leads to rapid and severe degeneration, whereas the needle puncture model results in mild and gradual degenerative changes, making it suitable for studying early stages of degeneration. Additionally, nucleotomy, which involves the removal of the NP, has been shown to cause significant structural damage, leading to rapid and pronounced degeneration [173]. A novel in vivo sheep model has also been developed to induce IDD by injecting 5-bromodeoxyuridine directly into the intervertebral disc using a percutaneous, CT-guided needle puncture method [174]. These models are highly reproducible and useful for various experimental studies.

Research is also conducted to assess spinal stability in sheep models by removing posterior structures, such as the lamina and LF, and evaluating changes in spinal stability during flexion/extension and torsion [175]. These studies demonstrate that posterior structures play a crucial role in spinal stability, and their removal can lead to spinal instability. Such research contributes to the development of reinforcement techniques to prevent complications, such as proximal junctional kyphosis, which may arise after surgical procedures [176]. In studies of disc regeneration, the injection of a cell-loaded collagen hydrogel into sheep intervertebral discs post-discectomy or partial nucleotomy, combined with nucleus augmentation and annulus repair, has been shown to promote disc regeneration [177,178]. This approach contributes to the development of regenerative treatments for degenerative disc disease. While sheep share structural and biomechanical similarities with human spines, their quadrupedal locomotion introduces differences in mechanical stress distribution that may not fully match human conditions. Therefore, direct application of these results to humans has limitations. Additional studies are needed to evaluate the long-term degenerative changes and regenerative capacities in sheep models.

The various methods used to create lumbar-level IDD models in large animals are summarised in the Table 6.

### 2.7. Comparative Cellular Phenotypes and Degenerative Progression in Rodent, Rabbit, and Canine IDD Models

Significant phenotypic differences in cells related to disc composition, cellular characteristics, and rate of pathological change are observed among rodents, rabbits, and canines in IVD disease research. Rodent IVDs are composed of structures similar to those in humans, including the NP, AF, and cartilage endplates (CEP) above and below, but they differ structurally due to their small disc size and the mechanical load associated with quadrupedal locomotion, which differs from that of human lumbar discs [209]. At the cellular level, rodent IVDs share many common features with human IVDs, such as decreased cell counts, apoptosis, aging, and increased immune cell infiltration during aging or induced degeneration [210,211]. However, the NP in rodents retains more notochordal cells compared to humans, resulting in a relatively slower degeneration rate [212,213]. Rodent discs are also highly responsive to damage or external stimuli, making them suitable for quickly observing degenerative changes [214]. Furthermore, genetic modification allows for the targeted removal of specific proteins, enabling a more detailed study of degenerative changes in rodent models.

Rabbits, with their similar disc structure to humans, are well-suited for studying mid- to late-stage disc degeneration. Like humans, rabbits experience anterior osteophyte formation as part of the disc degeneration process, although this typically occurs later in human disease progression [215,216]. Additionally, as rabbits age, there is a reduction in COL2A1 and AGC1 expression, while COL1A1, MMP-13, BMP-2, MGP, and p21 increase in NP cells, reflecting cellular changes similar to those observed in human disc degeneration [217].

Chondrodystrophic (CD) and non-chondrodystrophic (NCD) canines represent a unique model for studying human-like degenerative changes in the spine, exhibiting differences in disc composition, cellular characteristics, and the rate of pathological progression [218]. Both CD (e.g., Beagles, Dachshunds) and NCD canines possess a disc structure similar to humans with AF, NP, and CEP [212,219]. However, CD canines undergo rapid fibrosis in the NP, leading to early disc degeneration, making them suitable for modeling early-stage human IDD. In contrast, NCD canines show resistance to degeneration, maintaining disc structure for longer, thus serving as a model for studying gradual degenerative processes similar to those in humans [218,220].

On the cellular level, CD canines present fibrocartilaginous cells in the NP from an early stage, with rapid progression of degeneration as they age [221]. They exhibit increased activity of matrix metalloproteinases (MMPs), decreased glycosaminoglycan (GAG) content, reduced proteoglycan levels, and a shift from collagen type II to collagen type I in the NP, along with the formation of chondrocyte-like cells and an increase in inflammatory responses, including elevated prostaglandin E2 (PGE2) and COX-2 expression [222,223]. These characteristics resemble human IDD and mirror similar degenerative stages observed in MRI [223,224]. CD canines exhibit chondrification and fibrosis in the NP, showing high similarity to human models in terms of cellular phenotype and pathological features. In contrast, NCD canines retain notochordal cells into maturity, with stable proteoglycan and collagen II levels in the NP, demonstrating resilience to degeneration [212,225].

IVD disease in CD canines primarily occurs between the ages of 3 and 7 in the cervical (C2–C6) or thoracolumbar (T11–L3) regions, with progressive disc height loss, NP fibrosis, and AF damage, making them ideal for modeling early human disc degeneration [218]. In NCD canines, disc degeneration mainly occurs between the ages of 6 and 8 in the lumbosacral (L6–S1) or caudal cervical (C5–T1) regions, and they maintain disc structure for longer without external stimuli, making them suitable for studying gradual degenerative changes in humans [218,226,227].

Given these characteristics, CD canines, which show degenerative changes similar to humans, are valuable for studying human IDD due to their naturally occurring disc degeneration and comparable pathological features. While current treatments—such as physical therapy, medication, and surgery—provide symptomatic relief, they do not fundamentally restore disc structure. Therefore, research into regenerative therapies using canine models that closely mimic human degenerative changes is crucial in developing new, clinically applicable therapies for degenerative disc disease.

Promising regenerative treatments for degenerative IVD include cell-based therapies, growth factor applications, gene therapies, and NTG-101, a novel molecular therapy containing recombinant human (rh) transforming growth factor beta 1 (TGF-β1) and connective tissue growth factor (CTGF) [227,228]. Cell-based strategies using chondrocyte-like cells, mesenchymal stromal cells, and notochordal cells aim to regenerate damaged cells and tissues in degenerative discs. When combined with growth factors and gene therapy, these strategies can maximize the regenerative potential of IVD cells. These new therapeutic approaches focus on reconstructing the extracellular matrix and suppressing inflammation, even in advanced degenerative IVD stages, contributing to the control of IDD progression [227]. Therapies like NTG-101 can significantly aid in restoring biomechanical function and maintaining tissue homeostasis in IVDs through anti-inflammatory, anti-degenerative, and regenerative effects [228]. Such preclinical research holds promise for future clinical applications, and further studies using animal models that exhibit human-like degenerative changes, such as CD canines, could validate these therapies for clinical translation.

## 3. Advantages of Animal Models of Disc Diseases

Next, we discuss the advantages of using animal models in IVD disease research. Animal models provide a controlled and standardised environment for studying IVD diseases, which can help identify the underlying mechanisms of the diseases. Animal models also enable long-term monitoring of potential treatments, including new drugs, therapies, and surgical procedures, before application in humans. Moreover, animal models can provide important insights into the development and progression of the disease as well as the development of therapies through large-scale monitoring before clinical use. Here, we review the following four advantages of using animal models of disc diseases: controlled environment, long-term monitoring, controlled effect sample size for statistical significance, and cost-effectiveness.

### 3.1. Controlled Environment

IVD disease research in human patients is frequently limited by the inability to manipulate variables or achieve a controlled environment [229]. Human studies are usually observational, and the effects of different variables on disc disease cannot be controlled. An advantage of using animal models is that researchers can create a controlled environment to study the effects of specific variables on the development and progression of disc diseases and effects of potential treatments on disc health. Researchers can also manipulate environmental factors, such as age, diet, genetics, exercise, environment, and exposure to different substances, to create conditions that mimic the human disease state [230]. For example, researchers may create a controlled environment that mimics the effects of aging in the disc or induce inflammation in the disc by injuring it or exposing the animal to specific substances. Animal models thus serve as reliable and reproducible models for disc disease research in a controlled environment.

### 3.2. Long-Term Monitoring Investigation

Animal models also allow the study of the natural history of disc diseases; animals can be followed longitudinally throughout their lifespan, allowing the monitoring of disease progression over time [231]. Such longitudinal monitoring is challenging in human studies, as many variables influence disease progression in humans and ethical considerations limit the performance of invasive procedures. In contrast, animal models can be used to monitor the development of disc disease from its earliest to later stages. Notably, studying the progression of disc disease in animal models can provide insights into the underlying mechanisms of the disease, such as the molecular and cellular changes that occur as the disease progresses [214]. This information can be used to develop new treatments that target specific aspects of the disease or to identify biomarkers that can be used to diagnose the disease at an earlier stage [160]. Researchers can test the effectiveness of different therapies at different stages of disease progression and identify the most suitable treatment at each stage. A detailed understanding of disease progression and potential treatments at different disease stages can help improve patient outcomes and potentially arrest disease progression.

### 3.3. Controlled Effect Sample Size for Statistical Significance

Performing human studies with large sample sizes can be challenging because of limitations in recruiting patients with specific diseases or conditions. Additionally, the findings of human studies may be affected by confounding factors, making it difficult to draw conclusions regarding the underlying mechanisms of the disease and effectiveness of treatments. Studying disc diseases in animals enables researchers to control the environment and variables being studied and obtain a large volume of data on the disease and response to treatment. Furthermore, effect sample sizes in animal studies can increase the generalisability of these findings to human populations [232]. This is particularly important in medical research because animal models are frequently used to test the safety and efficacy of treatments before they are tested in humans. Using effect sample sizes in animal studies can increase the reliability of statistical tests and improve the accuracy of results [233]. It can help researchers obtain statistically significant results, reach robust conclusions regarding the effects of treatments and mechanisms of the diseases, and better predict the effects of the treatments in humans. This has important implications for developing new treatments and therapies to improve patient outcomes.

### 3.4. Cost-Effectiveness

Developing new treatments for disc diseases can be time-consuming and expensive, and using animal models can provide an efficient and cost-effective platform to investigate potential treatments [234]. Animal studies are generally less expensive and have shorter timelines than human clinical trials, enabling rapid evaluation of potential treatments before progression to human clinical trials. Furthermore, animal studies can be designed to investigate multiple treatments simultaneously, allowing cost-effective comparison of treatment outcomes. Animal models can also provide valuable preliminary data that can guide the design of future human clinical trials, thereby potentially reducing the overall drug development costs [235]. Moreover, these models allow for the investigation of potential treatments in a controlled environment, thereby reducing the variability associated with human studies. This can increase the validity and reliability of the results and potentially reduce the overall cost of drug development by reducing the risk of failure in clinical trials. Therefore, the cost-effectiveness of using animal models in disc disease research is a significant advantage. However, the ethical considerations and potential limitations of animal models should be carefully considered when using them in disc disease research.

## 4. Limitations of Animal Models of Disc Diseases

Although animal models have provided valuable insights into the pathophysiology and treatment of disc diseases, their use has some limitations. First, the anatomy and physiology of animal spines differ significantly from those of humans, hindering accurate extrapolation of the findings to humans. Second, the pathophysiology of disc diseases in animals may not entirely mirror that in humans. Third, animal models may not adequately mimic the environmental and lifestyle factors that contribute to disc diseases in humans, such as smoking and a sedentary lifestyle. Finally, ethical considerations limit the use of animal models, making large-scale studies challenging. Here, we discuss the four major limitations of using animal models of disc diseases: species differences, ethical concerns, lack of standardised protocols, and short lifespan.

### 4.1. Species Differences

Although animal models are useful for studying human diseases and provide valuable insights into disc disease pathology, treatment, and prevention, inherent differences exist between species that limit the applicability of findings from animal models to humans [236]. Factors affecting the development and progression of disc diseases, such as spinal anatomy, biomechanics, physiology, and the immune system and inflammatory response, differ between animals and humans [237]. This makes the extrapolation of findings from animal studies to human populations challenging. Therefore, researchers should use multiple animal models or combine animal models with other methods of investigation, such as in vitro human cell studies or clinical trials. Combining techniques could provide a more comprehensive understanding of the mechanisms underlying disc disease and the efficacy and safety of potential treatments, aiding in the clinical translation of findings. Species differences are an important limitation of animal models of disc diseases, as they affect the applicability of results to the human population. Therefore, researchers should carefully select animal models based on their similarities to human biology, use multiple approaches to validate their findings, and ensure the translation of the results to human diseases.

### 4.2. Ethical Concerns

Animal research is subject to strict ethical guidelines and regulations to ensure that the use of animals is justified, and that animals are treated humanely. Animal research raises ethical concerns, particularly regarding the potential pain and distress experienced by animals during surgical procedures and other interventions [238]. Therefore, the use of animals in research should be carefully considered, and alternative methods, such as in vitro or computer modelling, should be explored when possible. The use of animal models of disc diseases, in particular, raises ethical concerns, which can be considered a significant limitation of this approach [239]. According to the Institutional Animal Care and Use Committee protocol, animals used in disc disease models are classified as those subjected to experiments related to pain or distress using appropriate anaesthetics, analgesics, or sedatives corresponding to pain grade D [240]. A major ethical concern is the potential harm that may be caused to animals during the research. Depending on the animal model and research question, animals may be subjected to surgical procedures, behavioural tests, or other interventions that can cause pain, distress, or long-term health problems. Additionally, animal models may involve the use of anaesthesia, which carries its own risks. Another ethical concern involves the use of animals as surrogates for human diseases [241]. Although animal models can provide valuable insights into the mechanisms underlying disc diseases and potential treatments, animals are not perfect surrogates for human diseases. This raises questions about the justification for using animals in research, mainly if the research benefits are not immediately applicable to human health. Finally, the use of animal disc disease models raises broader ethical questions regarding the use of animals in scientific research. Some argue that the use of animals in research is inherently unethical, whereas others argue that it is ethical as long as it is conducted under strict guidelines and regulations. Overall, ethical concerns are a significant limitation to the use of animal models of disc diseases. Therefore, researchers should ensure that animals are treated humanely and that their suffering is minimised. This requires careful consideration of factors, including housing conditions, diet, and pain management, among others. The ethical use of animal models also requires the use of the minimal number of animals necessary to achieve the research goals as well as the use of alternatives to animal models, whenever possible. Additionally, researchers should carefully consider the limitations of animal models, use multiple approaches to validate the findings, and ensure the translation of the results to human diseases.

### 4.3. Lack of Standardised Protocols

One of the main disadvantages of disc disease animal models is the lack of standardised protocols. There is frequently significant variability in animal studies’ design, implementation, and analysis. Different researchers may use animals of various species, ages, and sex as well as other study designs, criteria for disease severity, methods for inducing disc disease or injury, outcome measures, and statistical analyses. Without clear and consistent methods for inducing disc disease or injury, assessing disc pathology, and measuring treatment effects, it can be challenging to determine the reproducibility of findings or validate the efficacy and safety of potential treatments. Induction methods that are not well established or have not been validated can increase the risk of harm to animals, raising ethical concerns regarding animal welfare [242]. The lack of standardisation can also make it challenging to compare findings between studies, determine the optimal animal model for a given research question, and translate results from animal models to human patients. Therefore, researchers should establish standardised protocols for animal models of disc disease to address these limitations. This can involve collaboration between researchers to develop a consensus on methods and outcome measures and efforts to validate findings across multiple animal models and human clinical trials.

### 4.4. Short Lifespan

Many animals commonly used in disc disease models have shorter lifespans than humans. For example, the average lifespan of mice, rats, and rabbits ranges from 2 to 4 years [243,244,245]. This limits their utility for studying the long-term effects of disc disease or evaluating the efficacy and safety of potential treatments over a relevant timescale. Additionally, animals may not develop the same age-related changes in the spine as humans, which can affect the relevance of animal study findings. The short lifespan of some animal models makes it difficult to study age-related disc degeneration, which is a common cause of disc diseases in humans. Although some animal models can be modified to mimic age-related degeneration, studying the effects of disc diseases over long timescales that are relevant to human health can remain challenging [246]. Therefore, the short lifespan of animals is a significant limitation in the use of animal models of disc diseases. Researchers should use multiple animal models with different lifespans to study the effects of disc diseases and potential treatments on relevant timescales to address these limitations. Additionally, researchers may use techniques, such as genetic modifications or diet manipulation, to induce age-related degeneration in animal models. Researchers should carefully select animal models and study designs to ensure that they are appropriate for the research question and relevant to human health.

## 5. Future Directions

To overcome the limitations of animal models of disc diseases, researchers should continue developing and refining animal models to resemble human conditions more closely. This can be achieved using larger animals, such as nonhuman primates, and by developing models that consider environmental and lifestyle factors. Additionally, researchers should continue to explore the mechanisms underlying disc diseases to identify new therapeutic targets. Finally, using advanced imaging techniques, such as MRI and computed tomography (CT), will enable researchers to study disease development and progression in animal models more accurately. Animal models of disc diseases are valuable tools for studying the underlying mechanisms and potential treatments for these conditions. However, there is still more to be learned, and the ongoing research focuses on several future directions, four of which are discussed below.

### 5.1. Development of More Representative Animal Models

As previously mentioned, there is currently no standardized protocol for developing animal models for spinal stenosis and disc diseases, which can lead to inconsistent results and make comparisons between studies challenging. The model used in this study, which induces spinal stenosis through implantable materials like silicone, provides a practical approach to replicate key pathological features, such as nerve root compression, tissue inflammation, neural damage, increased pain, and decreased motor function in the early to intermediate stages, within a short timeframe [13,14,15,16,17,18,19,20,21,22,23,24,25,26,27]. Although this model effectively simulates acute responses within a limited period, it does not fully capture the gradual progression of spinal stenosis observed in humans, which develops over time due to degenerative factors like disc disease, facet joint hypertrophy, and LF thickening. To more accurately reflect the pathophysiology and clinical progression of human spinal stenosis, future research should focus on developing animal models that incorporate gradual changes, such as progressive LF thickening. Additionally, using larger animals or developing models with genetic predispositions or environmental factors that increase vulnerability to spinal disc degeneration may enhance the clinical relevance of these models. Such advancements would contribute to more consistent data in spinal stenosis and disc disease research, ultimately enhancing the potential for clinical application.

### 5.2. Use of Advanced Imaging Techniques

Advanced imaging techniques, such as MRI, CT, and micro-CT, can provide detailed information regarding the structural and functional changes that occur in the spine during the development and progression of disc diseases [247,248]. This can improve our understanding of the disease mechanisms and aid the development of new treatments. These imaging techniques could enable better visualisation and characterisation of disc diseases in animal models. Thus, future research should focus on improving these imaging techniques to provide more detailed and accurate information on disc pathology in animal models.

### 5.3. Development of Non-Invasive Techniques

Current animal models of disc diseases frequently involve invasive surgical procedures that can be painful and distressing to the animals. Therefore, future research should focus on developing non-invasive treatments, such as pharmacological or cellular therapies, that can be tested in animal models before translation to human patients [249]. In addition, non-invasive techniques, such as ultrasound or optical imaging, should be applied to obtain information on the development and progression of disc diseases without the need for surgery [250].

### 5.4. Use of Genetic and Biomechanical Models

Advances in genetic engineering have enabled the development of animal models that mimic specific genetic mutations associated with disc diseases. In addition, biomechanical modelling can help simulate the mechanical loading and stresses on the spine during movement. Advances in genetic engineering have enabled the development of animal models that mimic specific genetic mutations associated with disc diseases. In addition, biomechanical modelling can help simulate the mechanical loading and stresses on the spine during movement. Therefore, future research should focus on constructing genetic models and biomechanical analyses to gain deeper insights into the underlying mechanisms of the diseases and the development of targeted therapies.

### 5.5. Advancements in AI-Driven Analysis of IVD

Artificial intelligence (AI) techniques are utilized extensively in analyzing various medical data, playing a crucial role in precision medicine for personalized treatment prediction, therapeutic efficacy evaluation, surgical outcome analysis, and the development of customized rehabilitation protocols [251,252,253]. A recent study analyzed 1000 fibroblast cells from Parkinson’s disease patients using AI, enabling an in-depth understanding of cellular structural changes and complex molecular events [254]. This study highlighted AI’s potential to aid in comprehending complex disease processes and supporting therapeutic development. In spinal diagnostics and treatment assessment, AI also enhances the accuracy and efficiency of standard imaging techniques, such as MRI and PET/CT, offering a more precise and standardized approach to spinal care [255]. This contributes not only to clinical diagnostics but also to research on IVD degeneration treatments. The introduction of AI into histopathological data evaluation allows for complex multi-tissue pathology assessment, analysis of various pain indicators, genomic data generation, and the application of advanced imaging techniques. This AI-based approach enhances the interpretation of research findings and facilitates their translation into human therapies. In the future, AI-driven technology is expected to improve the quality of animal model research, support understanding of IVD complexities, and aid in the development and evaluation of effective therapies.

## 6. Conclusions

Animal models have been instrumental in advancing our understanding of IVD diseases, such as lumbar spinal stenosis, disc herniation, and degeneration. These models offer insights into disease mechanisms and potential therapies by allowing for controlled experimental conditions and long-term monitoring. However, limitations remain, including species differences, ethical concerns, and the lack of standardized protocols. Future research should focus on developing more representative animal models, integrating advanced imaging and noninvasive techniques, and leveraging genetic and biomechanical modeling to enhance translational relevance. Additionally, AI-based analytics can improve model accuracy and precision, helping to develop more targeted, effective therapies for these debilitating conditions, with the potential to significantly enhance patient outcomes.

## Figures and Tables

**Table 1:** Lumbar spinal stenosis rodent model with silicone implants.

Authors	Animal Used	Sex	Age (Weeks)/ Weight (Gram)	Implanted Spine Level	Biomaterial	Manufacturing Company	Provided Characteristics
Watanabe et al. (2007) [27]	SD rats	M	200–250 g	L5	One silicone block		Length: 4 mm, width: 1 mm, thickness: 0.9 mm
Ito et al. (2007) [16]	SD rats	M	6 weeks/200–250 g	L5	One silicone block		Length: 4 mm, width: 1 mm, thickness: 1 mm
Sekido et al. (2012) [26]	Wistar rats	F	180–190 g	Between L5 and L6	One silicone rubber	Yamanaka Chemical Ind., Ltd., Osaka, Japan	Length: 3.5 mm; width: 5.0 mm; thickness: 0.5 mm
Shunmugavel et al. (2013) [20]	SD rats	F	225–250 g	L4 and L6	Two silicone blocks	Bentec Medical Inc., Woodland, CA, USA	Length: 4 mm, width: 1 mm, thickness: 1 mm
Li et al. (2013) [25]	SD rats	M	230–280 g	L5	Silica gel slices	C. R. Bard, Inc., Covington, GA, USA	Length: 4 mm, depth: 1.25 mm, thickness: 1 mm
Khan et al. (2015) [17]	SD rats	F	Approximately 300 g	L4 and L6	Two silicon blocks	Bentec Medical Inc., Woodland, CA, USA	Length: 4 mm, width: 1 mm, thickness: 1 mm
Uranbileg et al. (2019) [21]	SD rats	F	8–10 weeks/200–250 g	L3 and L5	Two silicon blocks	King Works Co., Ltd., Osaka, Japan	Length: 4 mm, width: 1 mm, thickness: 1 mm
Cheung et al. (2019) [22]	SD rats	F	13.0–14.5 weeks	L4 and L5	Two silicon sheets		Thickness: 0.51 mm
Park et al. (2019) [19]	SD rats	M	6 weeks/215.1 ± 9.7 g	Between L4 and L5	One silicone block	Bentec Medical Inc., Woodland, CA, USA	Length: 4 mm, width: 1 mm, thickness: 1 mm
Lee et al. (2019) [24]	SD rats	M	250–270 g	Between L5 and L6	Trapezoid-shaped silicone block		Length: 1 mm, width: 1.3–1.2 mm, height: 1 mm
Kim et al. (2021) [13]	SD rats	M	7 weeks/230–250 g	L4	One silicone block	KCC Corporation, Seoul, Korea	Silicone components (siloxanes, silicones, di-Me, vinyl groups, and silicone dioxide), mixing ratio, elastic modulus, stiffness (70, 80, and 90 kPa), size (length: 4 mm, width: 1 mm, thickness: 1 mm)
Lee et al. (2021) [23]	SD rats	M	250–270 g	Between L5 and L6	Trapezoid-shaped silicone block		Length: 1 mm, width: 1.3–1.2 mm, height: 1 mm
Hong et al. (2021) [14]	SD rats	M	7 weeks/230–250 g	L4	One silicone block	KCC Corporation, Seoul, Korea	80 kpa, length: 4 mm, width: 1 mm, thickness: 1 mm
Hong et al. (2022) [15]	SD rats	M	7 weeks/230–250 g	L4	One silicone block	KCC Corporation, Seoul, Korea	
Kim et al. (2022) [18]	SD rats	M	7 weeks/230–250 g	L4	One silicone block	KCC Corporation, Seoul, Korea	

Abbreviations: SD, Sprague Dawley rats; M, male; F, female; L3, lumbar 3.

**Table 2:** Methods for producing lumbar spinal stenosis model using rabbits.

Authors	Animal Species	Sex	Age (Weeks)/Weight (Gram)	Implanted Spine Level	Biomaterial	Manufacturing Company
C. Sun et al. (2021) [34]	Rabbit	F	≈3.0 kg	L2–L3, L4–L5	2 mm × 10 mm titanium locking screw	Watson locking plate; Changzhou, China
K. Hayashi et al. (2017) [33]		M	18 weeks/2800–3200 g	L2–L3, L4–L5	2.0 mm titanium locking screw	Universal Mandibular System; Leibinger, Stuttgart, Germany
H.I. Secer and Y. Izci (2008) [36]		M	14 weeks/1420–1570 g	L6	Two 3-F Fogarty catheters, balloons of catheters,	Pruitt-Nozick, Irrigation Embolectomy Catheter, Ideas for medicine Inc.
A. Yabu et al. (2023) [35]		M	18 weeks	L2–L3, L4–L5	2.0 mm titanium locking screw	Universal Mandibular System; Leibinger, Stuttgart, Germany

**Table 3:** Lumbar disc herniation rodent model with autologous NP transplantation.

Authors	Animal Used	Sex	Age (Weeks)/Weight (Gram)	NP Harvest Locations	Amount of NP Transplanted	NP Transplant Location
Obata et al. (2002) [41]	SD rat	M	200–250 g	Co	2 mg	Left L4 and L5 nerve roots
Cuellar et al. (2013) [43]	SD rat	M	300–400 g	Co		L5 DRG
Cho et al. (2013) [42]	SD rat	M	200–250 g	Co2, Co3		Left L5 DRG
You et al. (2013) [44]	SD rat	F	200–250 g	Co2, Co3		Left L5 DRG
Zhu et al. (2014) [46]	SD rat	M	200–250 g	Co2, Co3		Left L5 DRG
Han et al. (2015) [48]	SD rat	M	200–250 g	Co		Left L5 DRG
Wang et al. (2015) [52]	SD rat	M	220 ± 20 g	Co2, Co3	5 mg	Left L5 and L6 nerve roots
Kato et al. (2015) [50]	SD rat	F	200–250 g	Co		Left L5 DRG
Cho et al. (2015) [47]	SD rat	M	200–250 g	Co2, Co3		Left L5 DRG
Miao et al. (2015) [51]	SD rat		200–250 g	Co	10 mg	Right L5 nerve roots
Yan et al. (2015) [57]	SD rat	M	200–240 g	Co		Left L5 and L6 nerve roots
Liu et al. (2016) [55]	SD rat	M	220–250 g	Co2		Right L5 DRG
Cho et al. (2016) [53]	SD rat	M	200–250 g	Co2, Co3		Left L5 DRG
Cho et al. (2016) [54]	SD rat	M	200–250 g	Co2, Co3		Left L5 DRG
Yan et al. (2016) [56]	SD rat	M	220 ± 20 g	Co2, Co3	5 mg	Left L5 and L6 nerve roots
Yao et al. (2017) [63]	SD rat	M	200–250 g	Co2, Co3		Left L5 DRG
Seki et al. (2017) [66]	SD rat	F	200–240 g	Co		Left L5 DRG
Wang et al. (2017) [61]	Wistar rats	M	250–350 g	Co2	0.5 mg	Right L5 DRG
Wu et al. (2017) [62]	SD rat	M	250–300 g	Co	0.4 mg	Left L5 DRG
Yang et al. (2018) [67]	SD rat	M	300–400 g	Co1		L5 DRG
Huang et al. (2018) [64]	SD rat	M	260–300 g	Co2		L5 DRG
Zhong et al. (2018) [68]	SD rat		200–250 g	Co	10 mg	Left L4 and L5 nerve roots
Liu et al. (2019) [70]	Wistar rats	M	180–220 g	Co	5 mg	Right L5 DRG
Kaneuchi et al. (2019) [69]	SD rat	F	190–210 g	Co		Left L5 DRG
Zhong et al. (2019) [72]	SD rat		200–250 g	Co	10 mg	Left L4 and L5 nerve roots
Wang et al. (2019) [71]	SD rat	M	220 ± 20 g	Co2, Co3	5 mg	Left L5 and L6 nerve roots
Wang et al. (2020) [74]	SD rat	M	200–250 g	Co2	0.4 mg	L5 DRG
Yomogida et al. (2020) [75]	SD rat	F	8 weeks/150–180 g	Co		Left L5 nerve roots
Kwak et al. (2020) [73]	SD rat	M	200–250 g	Co2, Co3		Left L5 nerve roots
Zhu et al. (2021) [80]	SD rat	M	200–250 g	Co	10 mg	Left L5 nerve roots
Xu et al. (2021) [77]	SD rat	M	3 months/200–220 g	Co2, Co3		Left L5 and L6 nerve roots
Zhang et al. (2021) [78]	SD rat	M	200–220 g	Co		Left L5 nerve roots
Zhao et al. (2022) [79]	SD rat	M	260–300 g	Co2		Right L5 DRG
Qingguang et al. (2022) [82]	SD rat	M	200–250 g	Co	2 mg	Left L5 nerve roots
Xie et al. (2022) [76]	SD rat			Co2, Co3		Left L5 and L6 nerve roots
Li et al. (2022) [81]	SD rat	M	8 weeks	Co2	5 mg	Left L5 and L6 nerve roots

Abbreviations: Co, coccygeal; NP, nucleus pulposus; DRG, dorsal root ganglion.

**Table 4:** Methods for producing lumbar disc herniation model using large animals.

Authors	Animal Species	Herniated Location	Herniation Induction Method
M. Cornefjord et al. (2004) [90]	Pig	S1	(1) Application of the 100 mg of the harvested autologous NP from L2 to L3 disc; (2) Ameroid constrictor placement; (3) Combined application of autologous NP and ameroid constrictor.
T. Hasegawa et al. (2000) [93]	Dog	L5–L6, L6–L7	Placement of the tail AF and NP disc fragments in the anterolateral epidural space.
M. Sekiguchi et al. (2008) [91]		L6–L7	Puncture using an 18-gauge needle to allow leakage of NP into the epidural space near the nerve roots.
A. Minamide et al. (1998) [94]	Rabbit	L4	Placement of the L1–L2 disc fragments into the posterior epidural space at the L4 vertebra.
A. Minamide et al. (1999) [95]			
G. Zhou et al. (2010) [96]			

**Table 5:** Lumbar disc degeneration rodent model with ventral disc puncture.

Authors	Animal Used	Sex	Age (Weeks, or Months)/Weight (Gram)	Puncture Site	Puncture Tool	Puncture Depth	Von Frey Test	LBP Test
Lai et al. (2015) [112]	SD rats	M	-	L3–4, L4–5, L5–5	26 G needle	3 µm	Significance: 7 days after surgery	
Luan et al. (2015) [113]	SD rats	M	220–250 g	L5–6 (left transperitoneal)	26 G needle	Injected CFA		
Zhang et al. (2017) [114]	SD rats	M	8–9 weeks/200–250 g	L4–5	21 G needle	3 mm		
Fukui et al. (2017) [115]	SD rats	M	250 g	Bilateral L4–5 facet joint		Resection (lumbar facetectomy)		
Jiang et al. (2017) [116]	SD rats	M/F	6–8 months/250 g	L5–6	22 G needle	1–2 mm		
Maerz et al. (2018) [117]	Lewis rats	F	14 weeks/200 g	L3–4, L5–6	No.11 scalpel blade	1.5 mm		
Maas et al. (2018) [118]	Wistar rats	M	258 ± 7 g	L4–5	Tenotomy knife	2.5 mm		
Lai et al. (2019) [119]	SD rats	M	4–5 months	L3–4, L4–5, L5–6	26 G needle	3 mm	No significance	
Park et al. (2019) [120]	SD rats	M	3 months/350–400 g	L4–5	22 G needle	Suctioned NP	Significance: 7 days after surgery	
Long et al. (2019) [121]	SD rats	M	3 months/250–300 g	L4–5	21 G needle	3 mm		
Newton et al. (2019) [122]	Lewis rats	F	14 weeks/200 g	L3–4, L5–6	No.11 scalpel blade	1.5 mm		
NaPier et al. (2019) [123]	SD rats		3 months	L3–4, L5–6	18 G needle			
Mosley et al. (2019) [124]	SD rats	M/F	4 months	L3–4, L4–5, L5–6	26 G needle	3 mm, injected TNF-alpha		
Gao et al. (2019) [125]	SD rats		225–250 g	L5–6 (right side)	21 G micropuncture needle	2.3 mm		
Chen et al. (2019) [126]	SD rats	M				Removed vertebrae L1–6, T11–13		
Glaeser et al. (2020) [127]	SD rats	M	20 weeks	L3–4, L4–5, L5–6	18 G needle	2 mm		
Mosley et al. (2020) [128]	SD rats	M/F	4 months	L3–4, L4–5, L5–6	26 G needle	L3–4: male (42–46% of IVD height)/female (46–52% of IVD height) L4–5, L5–6: 40% of IVD height	Male: significant after 2 weeks Female: No significance	
Zhang et al. (2020) [129]	SD rats	M	8 weeks	L3–4, L4–5	28 G needle			
Cui et al. (2021) [130]	SD rats		9–10 weeks/250–300 g	L4–5	27 G needle			
Zheng et al. (2021) [131]	rats	M	250–300 g	L4–5	18 G needle	2 mm	Significance: 3 days after surgery	
Suh et al. (2021) [132]	SD rats	M	8 weeks/200–250 g	L4–5, L5–6	27 G Hamilton syringe		No significance	Rearing test
Lei et al. (2021) [133]	SD rats	M	6–8 weeks/250 ± 20 g	L3–4, L4–5, L5–6	21 G needle	2.3 mm		
Hong et al. (2022) [102]	SD rats	M	7 weeks/230–250 g	L4–5, L5–6	22 G needle	4 mm, suctioned NP	No significance	Tail suspension test
Chen et al. (2022) [134]	SD rats	M	200–250 g	L4–5	21 G needle	3 mm		
Liu et al. (2022) [135]	SD rats	M	8 weeks	L5–6	21 G needle	2.3 mm		
Tang et al. (2022) [136]	SD rats	M	6–8 weeks/200–220 g	L4–6 (upper and lower cartilage endplate)	21 G micropuncture needle	Whole layer of the annulus fibrosus		
Lillyman et al. (2022) [137]	SD rats	F	15 weeks	L5–6	Stainless steel dissection needle (Roboz, RS-6066, 10 µm tip)	3 mm (scrape one and six times)	No significance	
Tan et al. (2023) [138]	SD rats	M	3 months	L4–5	21 G needle			
Chai et al. (2023) [139]	SD rats	M	6 weeks/200 ± 10 g	L3–4, L5–6	21 G micropuncture needle	Entire annulus fibrosus		
Lai et al. (2023) [140]	SD rats	M	5–6 months	L4–5, L5–6	26 G needle	3 mm	No significance	

Abbreviations: LBP, low back pain; TNF, tumor necrosis factor.

**Table 6:** Methods for producing lumbar disc degeneration model using large animals.

Authors	Animal Species	Degenerated Location	Degeneration Induction Method
M. Pfeiffer et al. (1994) [152]	Pig	Lumbar discs	(1) Anterolateral nucleotomy with rongeurs after AF fenestration. (2) Nucleotomy with rongeurs, 5 mg hyaluronic acid/sodium salt solution instilled, closure with fibrin glue. (3) Chemonucleolysis with 1000 I.U. chymopapain (0.5 mL). (4) Anterior semicircular anulus dissection with lancet, no NP removal.
A. Baranto et al. (2005) [145]		L2-L4	Retroperitoneal approach, 3.5 mm hole in L2, L3, or L4 vertebrae, directed 45° cranially to endplate and NP.
J. Salo et al. (2008) [153]		L3-L4	L4 vertebra perforated with 3.5 mm drill bit, angled at 45°, hole drilled into NP.
H.B. Henriksson et al. (2009) [148]		L1-L6	NP aspirated with 20-gauge needle after 5 cm paravertebral incision and retroperitoneal dissection under full visual control.
G.W. Omlor et al. (2012) [151]		L2-L6	Partial nucleotomy in general anaesthesia by a 16 G biopsy cannula (removal of 0.04–0.12 g jelly-like nucleus).
H. Hebelka et al. (2013) [147]		L2-L3	3.5 mm drill, hole at mid-height L3 vertebra, 45° angle, penetrates central cranial endplate.
R. Kang et al. (2014) [149]		disc 4 or 5	No. 23 scalpel at the left anterolateral part at a depth of 6 mm.
G.W. Omlor et al. (2018) [150]		L1-L6	Four discs (L2–L5) with annulus puncture and partial nucleotomy.
C.H. Flouzat-Lachaniette et al. (2018) [146]		Three randomised lumbar discs	(1) AF punctured with 1.3 × 88 mm/18G × 3 needle. (2) Caudal endplate drilled with 3.5 mm bit, 45° angle, targeting NP centre. (3) Cryoinjury with 2.5 J Thompson cryoprobe, two cycles of 120 s, 1 min warming between cycles.
C. Techens et al. (2020) [154]		T13-L6	Square incision in AF with scalpel, NP extracted through excision with curette.
W.C. Hutton et al. (2000) [159]	Dog	L3-L4	Stainless steel springs across L3/L4, exerting compressive forces ranging from 92 to 156N.
C. Chen et al. (2015) [155]		L3-L6	18-gauge needle inserted through AF into NP centre, punctures limited to 7 mm depth with 11-scalpel blade.
Z. Shi et al. (2015) [158]		L1-L6	20-gauge biopsy gun (Cook; Quick-Core) percutaneously punctured into NP area via left posterior route.
T. Nukaga et al. (2016) [156]		L2-L5	18-gauge needle with stopper inserted through fibrous ring into NP centre, suction performed once with 10 mL syringe.
I. Rudnik-Jansen et al. (2019) [157]		T13-L4	18G needle inserted in outer AF, part of NP aspirated with 10 mL syringe.
Y. Aoki et al. (2006) [179]	Rabbit	L2-L5	18-gauge needle puncture with depth controlled at 1 mm or 5 mm using handmade stopper.
T. Chujo et al. (2006) [180]		L2-L5	Annular puncture model with 18-gauge needle, defined depth of 5 mm.
M.H. Kong et al. (2008) [181]		L1-L5	16-gauge needle punctured left anterolateral AF to 5 mm depth.
E. Vialle et al. (2009) [182]		L2-L7	Intervertebral space assessed, puncture areas (1–2 mm diameter) exposed, punctured three times by 18G needle at exact 5 mm depth.
F. Mwale et al. (2011) [183]		L2-L5	Annular puncture with 18-gauge needle, 5 mm depth on two non-contiguous discs.
D.D. Chan et al. (2011) [184]		L4-L5	16-gauge needle puncture under aseptic conditions, segments trimmed to include disc and approximately 5 mm.
C.H. Moon et al. (2012) [185]		L2-L5	16-gauge hypodermic needle used to puncture outer 5 mm of L2–3, L3–4, and L4–5 discs.
H.J. Chun et al. (2012) [186]		L2-L5	19-gauge spinal needle inserted into discs via posterolateral approach, 2 cm from midline spinous process, 30–45° angle from midline to lateral.
Y.J. Kwon (2013) [187]		L2-L5	18G angiography needle inserted 3–4 cm ventral from midline.
S. Ren et al. (2013) [188]		L3-L6	24-gauge needle used to pierce exposed intervertebral disc centre from side front, depth controlled at ~5 mm and held for 5 s.
I.L. Moss et al. (2013) [189]		L4-L5	Punctured to 5 mm depth with 18 G needle and rotated 360°, with suction applied through 10 mL syringe for 10 s.
H. Mao et al. (2014) [190]		L2-L5	NP tissues aspirated from three consecutive lumbar IVDs using 18 G needle and 5 mL syringe.
Y. Liu et al. (2016) [191]		L3-L6	Punctured with a 16-gauge needle to a depth of 5 mm.
X. Bai et al. (2017) [192]		X	Rabbit placed in upright posture in plastic tube for 4–6 h daily, with a 600 g Styrofoam and plummets collar on neck.
L. Xin et al. (2017) [193]		L3-L6	(1) Annular defects (1.8 mm diameter, 4 mm depth) created with mini-trephine. (2) Implantation group: defects filled with PLGA/fibrin gel plug. (3) Puncture group: 16 G needle created annular puncture, 5 mm depth.
Y. Wang et al. (2017) [194]		L2-L5	Injection needle (size 7) used for annulus puncture.
A. Calvo-Echenique et al. (2018) [195]		L4-L5	18-gauge needle used for disc injury at L4–L5, postero-lateral approach, 30–35 mm right to midline spinous process, angle 35–40° to horizontal plane.
T. Ishikawa et al. (2018) [196]		L2-L5	Annular punctures at L4/5 using 18-gauge or 21-gauge needle.
H.M. Xu et al. (2019) [197]		L4-L5	Two 2 cm incisions over L4–5 lumbar vertebrae to expose L4–5 vertebral body, stainless steel external shear loading device fixed in L4–5 vertebral body.
K. Sheldrick et al. (2019) [198]		Lumbar discs	18-gauge needle puncture.
Z. Zhao et al. (2020) [199]		L3-L7	24-gauge needle pierced into centre of exposed lumbar discs (~5 mm), 20 μL of PBS or 107 vp lentivirus injected into NP tissue.
B.G. Ashinsky et al. (2020) [200]		L2-L7	Punctured with a 16 G needle.
Y. Wang et al. (2021) [201]		L3-L5	(1) Percutaneous puncture with 18-gauge needle into disc centre, rotated 360°, held for 30 s at 5 mm depth. (2) Withdrawal of syringe for 10 s after puncture to aspirate part of NP
T. Sudo et al. (2021) [202]		L1-L6	MIA (0.01, 0.1, 1 mg) injected into rabbit IVDs posterolaterally to centre percutaneously using micro syringes, 31 G needle.
L. Hei et al. (2021) [203]		L3-L4	18 G needle for percutaneous puncture, tilted 15–25° ventrally, at L3–4, space 1.5 finger-widths right of spinous process, negative pressure maintained for 15 s with 5 mL syringe.
D. Dumanlıdağ et al. (2021) [204]		L4-L7	Percutaneous annular puncture with 18-gauge angiography needle under C-arm guidance.
T. Hasegawa et al. (2023) [205]		L2-L5	12.5 mU/10 μL pharmaceutical-grade condoliase injected with micro-syringes, 30-gauge microneedle.
Y. Jin et al. (2023) [206]		L2-L5	Layered tissue incision, retroperitoneum and adipose tissues exposed, paraspinal fasciculus psoas major muscle blunt dissected.
S.U. Chengguo et al. (2023) [207]		L2-L5	Anulus punctured with 18 G needle, 5 mm depth, held for 5 s.
J. Guo et al. (2023) [208]		L4-L5	Device externally placed, attached to two K-wires (diameter, 2.0 mm) using variable-speed electric drill, axial compression stress of 100 N applied
Xi et al. (2013) [166]	Monkey	L1-L2 to L5-L6	Disc puncture with 15 or 20 G needle.
Wei et al. (2014) [167]		L3-L4, L5-L6	1.5 mm diameter, 15 mm depth hole drilled in intervertebral body above/below disc; 2.0 mL bleomycin (1.5 mg/mL) injected per hole
Wet et al. (2015) [168]		L3-L4, L5-L6	1.5 mm diameter, 15 mm depth hole drilled in intervertebral body above/below endplate; 2.0 mL pingyangmycin (1.5 mg/mL) injected per hole.
Zhou et al. (2007) [174]	Sheep	L2–L3, L4–L5, L6–L7	Under CT guidance, an 18-gauge, 8.5 cm spinal needle was positioned at the center of the disc, and 800–1000 µL of BrdU (5 mg/mL) was injected.
Lim et al. (2017) [172]		L2-L3, L3-L4	3.5 mm Brad point drill bit to 12 mm depth.
Daly et al. (2018) [171]		L2-L3, L3-L4	(1) 3 mm × 5 mm annular window was created, disc resected with pituitary rongeurs; (2) 3.5 mm Brad point drill bit to 12 mm depth.
Sloan Jr et al. (2020) [177]		L1-L2 to L5-L6	Discectomy
Friedmann et al. (2021) [178]		Lumbar discs	Partial nucleotomy
Constant et al. (2022) [173]		L1-L2 to L3-L4	Three types of AF defects (slit, cruciate, and box-cut) created with standardized removal of 0.1 g NP at three lumbar levels (L1-L2, L2-L3, and L3-L4)

Abbreviations: PLGA, Poly(lactic-co-glycolic acid).

## Data Availability

The data presented in this study are available upon request from the corresponding author.
